# Effect of TiO_2_ Nanotube Pore Diameter on Human Mesenchymal Stem Cells and Human Osteoblasts

**DOI:** 10.3390/nano10112117

**Published:** 2020-10-25

**Authors:** Juan Shong Khaw, Christopher R. Bowen, Sarah H. Cartmell

**Affiliations:** 1 Department of Materials, The University of Manchester, Manchester M13 9PL, UK; jskhaw2020@gmail.com; 2 Department of Mechanical Engineering, University of Bath, Bath BA2 7AY, UK; C.R.Bowen@bath.ac.uk

**Keywords:** nanotube, pore diameter, surface nanotopography, stem cell osteogenesis, osteogenic differentiation, osteoblastic maturation

## Abstract

The pore diameter of uniformly structured nanotubes can significantly change the behaviour of cells. Recent studies demonstrated that the activation of integrins is affected not by only the surface chemistry between the cell-material interfaces, but also by the features of surface nanotopography, including nanotube diameter. While research has been carried out in this area, there has yet to be a single systemic study to date that succinctly compares the response of both human stem cells and osteoblasts to a range of TiO_2_ nanotube pore diameters using controlled experiments in a single laboratory. In this paper, we investigate the influence of surface nanotopography on cellular behaviour and osseointegrative properties through a systemic study involving human mesenchymal stem cells (hMSCs) and human osteoblasts (HOBs) on TiO_2_ nanotubes of 20 nm, 50 nm and 100 nm pore diameters using in-vitro assessments. This detailed study demonstrates the interrelationship between cellular behaviour and nanotopography, revealing that a 20 nm nanotube pore diameter is preferred by hMSCs for the induction of osteogenic differentiation, while 50 nm nanotubular structures are favourable by HOBs for osteoblastic maturation.

## 1. Introduction

Implant loosening and bone resorption are the most reported complications in orthopaedic implantations, therefore implants with improved osseointegration and increased longevity are continually sought after. The process of surface modification has the potential to enhance structural and functional connections between the bone-implant interfaces, and consequently improve the long-term performance of implants, while the desirable bulk attributes of the biomaterials are retained [1,2]. Surface modification can also offer better wear resistance, corrosion resistance, biocompatibility and surface wettability [3,4,5]. 

Nanostructured biomaterials, typically within the dimensional range up to approximately 100 nm, have received much attention due to their large surface area and increased biological responses in comparison to micro- and macro-surface structured materials and surfaces [6,7]. Electrochemical anodisation has been widely recognised as a reliable technique in generating TiO_2_ nanotubes on titanium-based biomaterials due to its simplicity, controllability and reproducibility. Together with optimised process parameters, this technique allows the formation of highly ordered nanotube arrays of controlled nano-scale dimensions for effective biological assessment [8,9,10]. 

TiO_2_ nanotubes formed on the surface of titanium and titanium alloys provide a unique surface nanotopography that gives rise to improved cell adhesion, proliferation and differentiation [11]. Many researchers in this area have demonstrated effective osseointegration properties on anodised titanium surfaces for nanotubes in the range of 10 to 100 nm in diameter [12,13,14]. The literature suggests that cell lines and cell species can affect cell performance and behaviour in response to the nanotopography of a surface, and this is due to small differences in the transmembrane receptors and their varied reactions to the extracellular matrix [15,16]. However, there remains a need for a deeper understanding of cell bioactivity events towards different nanotube diameters.

While there is existing data presented in the literature on understanding the influence of nanotube diameter, comparatively little effort has been undertaken to fully understand the influence of pore diameter on cellular activities. Nanotube pore diameter is often taken as a parametric dimension for cell adhesion, whereas the nanotube length does not influence cell behaviour in a significant way [17]. Previous work determining the relationship between nanotube diameter and mesenchymal stem progenitor cells suggested that a nanotube diameter below 30 nm led to enhanced mesenchymal osteogenic ability [14,17,18,19,20,21,22], with a small number of reports proposing that 70 nm to 100 nm is more favourable for mesenchymal stem cells [14,23,24], and a single study that has reported that 30 and 50 nm stimulated early osteoblastic gene expression [25]. However, for in-vitro osteoblastic cell lines studies, greater bone mineralisation has been demonstrated on the surfaces of nanotubes with a diameter range of 30 to 50 nm [26,27,28,29,30], while others have suggested that nanotube diameters of 15 to 30 nm displayed higher cell viability and proliferation [13,31,32]. Other published works comparing the unilateral nanotube samples to that of non-anodised samples indicated that 70 to 80 nm exhibited greater osteogenic ability [33,34,35], but those results are not adequate as a more extensive sample size should be included to obtain a more precise conclusion. Overall, there seems to be some evidence to indicate the precise range of nanotube diameters that are preferred by different cell lines, and it is preliminarily hypothesised that the more mature the cell phenotype, the larger the nanotube diameter preferred by the cells.

This research investigates the influence of surface nanotopography on cellular behaviour and osseointegrative properties through a systemic study involving human mesenchymal stem cells and human osteoblasts on TiO_2_ nanotubes with pore diameters of 20 nm, 50 nm and 100 nm. By conducting in-vitro testing in a single laboratory using anodised substrates produced from the same source, the potential for changing and limiting variables can be eliminated to allow an appropriate comparison between the performances of the two cell lineages so that the hypothesis could be validated more precisely and conclusively. The underlying cellular mechanism can also be elucidated once the data, that is currently not available in the literature, is produced. The knowledge gained in this study will enable essential comprehension of the cell-nanotopography biological interactions and promotes new approaches to tailor cellular osteogenic behaviour through controlled surface topography.

## 2. Materials and Methods

### 2.1. Sample Preparation and Characterisation

Pure titanium foils (thickness 0.25 mm; purity 99.5%; Alfa Aesar, Haverhill, MA, USA) were cut into a Ø14 mm disc shape using an AgieCharmilles CUT 200 Sp EDM wire-cut machine (GF Machining Solutions Management SA, Geneva, Switzerland). Before electrochemical treatment, the titanium foils were ultrasonically degreased in equal volumes of acetone, ethanol and deionised (DI) water for 5 min, followed by drying under a cool air stream. An anodising device with a three-electrode configuration was used. Titanium discs served as the working electrode, and a platinum foil (thickness 0.1 mm; purity 99.99%; Alfa Aesar, Ward Hill, MA, USA) served as the counter electrode, which was placed at a 40 mm distance from the working electrode. A saturated calomel Hg/Hg_2_Cl_2_ (1 M KCl) electrode was used as a reference electrode, connected to the anodisation setup by a salt bridge placed close to the working electrode. A mixture of 1 M H_3_PO_4_ and 0.25 wt% HF was used as the electrolyte.

Constant potentials were applied using an EG & G Instruments Scanning Potentiostat (Model 362, Princeton Applied Research, Oak Ridge, TN, USA) connected to a Ministat MKIV Sycopel Scientific signal amplifier (Sycopel Scientific Ltd., Tyne and Wear, UK). The anodisation experiments were carried out at room temperature for 60 min. Anodising potentials of 2.5 V generated TiO_2_ nanotube arrays with a pore diameter of 20 nm on the titanium disc, while 10 V and 20 V generated uniformly vertical nanotube array with pore diameters of 50 nm and 100 nm, respectively, on the titanium discs. In the following part, these anodised samples are labelled with respect to their nanotube pore diameter, i.e., Ti-20, Ti-50 and Ti-100. After the electrochemical treatment, the samples were rinsed with DI water and then ultrasonically cleaned for 10 min and further dried under a cool air stream. 

Structural and morphological characterisation of the nanotubes were undertaken using top-view images of anodised samples taken by a ZEISS Ultra 55 field emission scanning electron microscope (FE-SEM) (Carl Zeiss Microscopy GmbH, Jena, Germany), as reported previously [36]. The FE-SEM was operated under InLens detector mode with 5 kV EHT. ImageJ image analysis software (Version 1.51, National Institutes of Health, Kansas City, MO, USA) was used to measure the pore diameters of the anodised nanotubes formed. 

Water contact angle analysis was carried out using a CAM 200 contact angle measurement instrument (KSV Instruments Ltd., Helsinki, Finland). Using a precision syringe (Hamilton Threaded Plunger 1700 and 1000 Series Gastight Syringes (Hamilton Company Inc., Reno, NV, USA)), a droplet of deionised water measured was formed at the end of the blunt needle until the drop detached itself onto the surface of the sample. The contact angle was measured after the drop had come to rest (approximately 2 s), similar to the previously reported study [37]. Three drops on different areas of each sample were measured for consistency.

### 2.2. Cell Culture

Human mesenchymal stem cells (hMSCs) from bone marrow were obtained from (Hidalgo PT-2501, Lonza Group AG, Basel, Switzerland). Human osteoblasts (HOBs) were acquired from the cancellous bone of a 45-year-old male patient (C-12720 PromoCell GmbH, Heidelberg, Germany). Frozen vials of cells were thawed, cultured and expanded to reach the desired confluency based on the instructions provided by the company. For hMSCs, cells of passage 8 were prepared in growth medium (GM) consisting of Dulbecco’s Modified Eagle’s Medium (DMEM) with 4500 mg/L glucose, L-glutamine, and sodium bicarbonate, without sodium pyruvate, supplemented with 10% fetal bovine serum (FBS) and 1% Penicillin/Streptomycin (10,000 units/mL) all obtained from (Sigma-Aldrich, Merck Life Science UK Ltd., Gillingham, UK). For HOBs, cells of passage 5 were prepared in Osteoblast Growth Medium (PromoCell GmbH, Heidelberg, Germany). All the cells were maintained at 37 °C and 5% CO_2_ in a humidified incubator. 

The anodised titanium samples were designed with a tag to assist handling of samples using tweezers, which could be folded into L-shaped to ease the transference of samples without damaging the living cell culture during in-vitro experiments. Before cell seeding, a water-repellent circle was drawn around the top edge of the samples using a PAP pen (Thermo Fisher Scientific Inc., Waltham, MA, USA) to keep the cell seeding fluid pooled in a single droplet on the surface of anodised titanium samples. The samples were washed three times of 15 min each cycle with 70% ethanol to disinfect the sample and remove any impurity on the sample surface. All the titanium samples were then decontaminated in preparation for cell culture by exposing to ultraviolet (UV) light for 45 min each side. 

For hMSCs cell culture, 100 µL of cell fluid containing 1 × 10^4^ cells were seeded on each of the titanium samples. After one hour of cell seeding, GM was added to each well carefully from the wall of the well plate to minimise the turbulence of medium in the well. One day after the initial cell seeding (termed Day 1), half of the samples was refreshed with GM and another half with osteogenic medium (OM), prepared by adding 50 mg/mL ascorbic acid, 10 mM β-glycerophosphate and 10 nM Dexamethasone (Sigma-Aldrich, Merck Life Science UK Ltd., Gillingham, UK) to the GM. For HOBs cell culture, 100 µL of cell fluid containing 2 × 10^4^ cells were seeded on each of the titanium samples. Osteoblast Growth Medium was topped into each well carefully after one hour of cell seeding. The seeded samples were replaced with the fresh medium one day after the cell settlement. All the media were changed every three days.

### 2.3. AlamarBlue^TM^ Assay

To assess the cellular metabolic activity, the samples were assayed at Day 7 and 14 using AlamarBlue^TM^ assay. Briefly, 300 µL AlamarBlue^TM^ mixture containing 10% AlamarBlue^TM^ stock solution (Thermo Fisher Scientific Inc., Waltham, MA, USA) and 90% cell culture medium (GM for hMSCs and Osteoblast Growth Medium for HOBs) was added directly to the samples in a 24 well plate, then incubated at 37 °C for 60 min. Following incubation, three 50 µL replicates of the aliquots were transferred from each sample into a clear 96 well plate. Fluorescence readings were taken using a FLUOstar OPTIMA microplate reader (BMG Labtech GmbH, Ortenberg, Germany) at 560 nm excitation, 590 nm emission. After the assay, the samples were washed with fresh media and replaced with respective media (GM, OM or Osteoblast Growth Medium). The same samples were measured at each time point.

### 2.4. LIVE/DEAD Cell Viability Assay

Cell viability and morphology on the titanium samples were assessed by a fluorescence-based LIVE/DEAD cell viability assay (Thermo Fisher Scientific Inc., Waltham, MA, USA). The assay was performed at Day 1, 7 and 14. 4 mM calcein-AM in anhydrous dimethyl sulfoxide (DMSO) and 2 mM ethidium homodimer-1 in DMSO/H_2_O 1:4 (*v*/*v*) in phosphate-buffered saline (PBS) was added to each sample and incubated for 30 min, washed again with fresh PBS, and visualised using fluorescence light microscopy (Nikon Eclipse 50i (Nikon Corporation, Tokyo, Japan) equipped with Lucia GF-DXM1200 imaging software (Version 4.82, Lucia GF, Nikon Corporation, Tokyo, Japan)).

### 2.5. Cell lysate Preparation

Cell lysis was performed to allow the extraction of intracellular contents such as proteins, lipids, and nucleic acid. At Day 14, the samples in a 24 well plate were washed twice with PBS before a 300 µL of Gibco^®^ EDTA-free 0.25% Trypsin solution (Thermo Fisher Scientific Inc., Waltham, MA, USA) was added to each sample and incubated for 4 min. Mild vortex and gentle tapping were performed following incubation to ensure that the cells were detached from the samples. The trypsinised cells were transferred to a 1.5 mL microcentrifuge tube. 300 µL of GM was added twice to the well plate to wash and collect the leftover cells, then transferred to the same microcentrifuge tube and centrifuged at 300× *g* for 5 min. Following centrifuge, the supernatant was removed, and 250 µL of ALP assay buffer (BioVision Inc., Milpitas, CA, USA) was added to the cell pellet with an addition of the high-speed vortex to lyse the cells. The cell lysate was kept at −70 °C for further use.

### 2.6. PicoGreen DNA Quantification Assay

The DNA content on the samples was quantified using Quant-iT™ PicoGreen™ dsDNA Assay Kit (Thermo Fisher Scientific Inc., Waltham, MA, USA). The collected cell lysate was centrifuged at 13,000× *g* for 3 min before the assay. An optimal volume of cell lysate was added to the labelled clear 96 well plate, followed by an additional aliquot of 1x TE buffer to bring to a total volume of 100 µL. Another 100 µL of PicoGreen solution was added to the mixture to activate the reaction. The 96 well plate was protected from light and incubated for 5 min at room temperature. Subsequently, the fluorescence readings were taken using a FLUOstar OPTIMA microplate reader (BMG Labtech GmbH, Ortenberg, Germany) at 485 nm excitation, 535 nm emission. The DNA concentration was calculated according to the standard curve prepared as described in the manufacturer’s instruction.

### 2.7. Alkaline Phosphatase (ALP) Assay

The level of alkaline phosphatase (ALP) activity indicates an early marker of osteogenic differentiation. Before performing the ALP assay, the cell lysate was centrifuged at 13,000× *g* for 3 min to remove insoluble material. The ALP colourimetric assay kit (BioVision Inc., Milpitas, CA, USA) was used to measure the ALP activity in the samples. The *p*-nitrophenyl phosphate (*p*NPP) working solution was prepared according to the manufacturer’s instruction. ALP assay buffer and stop solution were readily prepared in the assay kit. To begin with the assay, an optimal volume of cell lysate was aliquoted to a clear 96 well plate in duplicate and the ALP assay buffer was added to bring the total volume to 80 µL. 20 µL of stop solution was added to one of the duplicated lysate mixtures (as background reading) to terminate the ALP activity. 50 µL of 5 mM *p*NPP solution was added to all the mixtures and incubated for 60 min at room temperature protected from light. The reaction was stopped by adding 20 µL of stop solution to the reacted samples (as sample reading), making a total volume of 150 µL in each well. The absorbance readings were taken using a Multiskan Ascent Microplate Reader (LabSystems Inc., Philadelphia, PA, USA) at 405 nm. The actual absorbance reading of the sample was obtained by subtracting the background readings from the sample readings. Subsequently, the ALP activity of the samples was calculated using Equation (1) provided by the manufacturer, where A represented the amount of *p*NP generated by the samples in µmol, V represented the volume of sample added in the assay well in mL, and T represented the reaction time in minutes. Normalisation of ALP level was tabulated by dividing the DNA concentration obtained from the PicoGreen assay.

ALP activity (U/mL) = A/V/T(1)

### 2.8. Immunofluorescence Staining

After cells were fixed using 10% formalin solution (Sigma-Aldrich, Merck Life Science UK Ltd., Gillingham, UK) for 15 min at room temperature, they were washed three times with PBS and permeabilised with 0.5% Triton X-100 and 0.05% Tween-20 in PBS for 10 min at room temperature. The permeabilised cells were then blocked with Immunocytochemistry (ICC) buffer consisted of 2% goat serum, 1% bovine serum albumin and 0.1% gelatin in PBS (Thermo Fisher Scientific Inc., Waltham, MA, USA) for 30 min at room temperature. The ICC buffer was later replaced by the primary antibody solution comprised of 1:400 mouse anti-vinculin (Thermo Fisher Scientific Inc., Waltham, MA, USA) in ICC buffer for 1 h, and washed with wash buffer (0.005% Tween-20 in PBS), followed by a secondary antibody solution consisted of 1:500 Alexa Fluor^®^ 568 Goat anti-Mouse (Thermo Fisher Scientific Inc., Waltham, MA, USA) and 1:1000 Alexa Fluor^®^ 488 Phalloidin (Thermo Fisher Scientific Inc., Waltham, MA, USA) in wash buffer for another 1 h. After washing twice with wash buffer, the samples were carefully mounted with ProLong^®^ Gold Antifade Mountant with DAPI (Thermo Fisher Scientific Inc., Waltham, MA, USA) to stain the cell nucleus. The samples mounted on glass slides and coverslips were sealed with transparent nail polish for microscopy imaging using a fluorescence light microscopy (Nikon Eclipse 50i (Nikon Corporation, Tokyo, Japan) equipped with Lucia GF-DXM1200 imaging software (Version 4.82, Lucia GF, Nikon Corporation, Tokyo, Japan)).

### 2.9. Data Presentation and Statistical Analysis

In this study, AlamarBlue^TM^ data was compared between Day 7 and Day 14. LIVE/DEAD data were compared between Day 1, Day 7 and Day 14 for better visualisation of cell morphology. All DNA content, ALP activity and immunofluorescence microscopy were conducted on Day 14 only due to the relevance of these tests are more substantial after 14 days of incubation. Statistical significance was analysed using the two-tailed paired Student’s *t*-test in Microsoft Excel (Version Excel 2016, Microsoft Corporation, Washington, DC, USA). Data are presented as mean ± standard deviation (S.D.). Probability (*p*-value) less than 0.05 was considered to be significant. 

## 3. Results

### 3.1. Surface Characterisation

**Figure 1:**
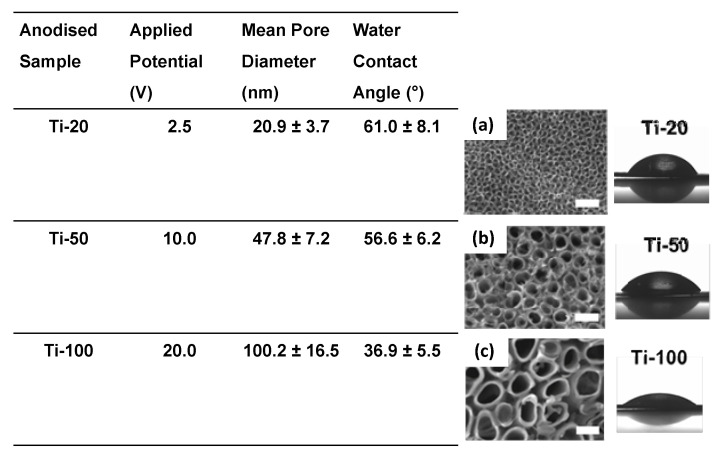
The applied voltage used for anodising pure titanium discs and the respective nanotube pore diameter and water contact angle with (**a**–**c**) top view FE-SEM micrographs of the anodised samples and their respective water contact angle diagrams. Scale bar = 100 nm.

The surface of pure titanium after anodisation treatment at the applied potentials listed in Figure 1 create a uniform array of nanotube layer, where a higher anodisation potential leads to larger nanotube pore diameters. The samples were labelled according to their regular pore diameters, for instance, Ti-20 represented anodised titanium samples with TiO_2_ nanotube of approximately 20 nm pore diameter, as shown in Figure 1a. As the nanotube pore diameter increased, the surface wettability also increased, as indicated by the decrease in contact angle in Figure 1. The most elevated hydrophilic character was observed on anodised titanium samples with 100 nm pore diameter (Figure 1c).

### 3.2. Human Mesenchymal Stem Cells Responses

**Figure 2:**
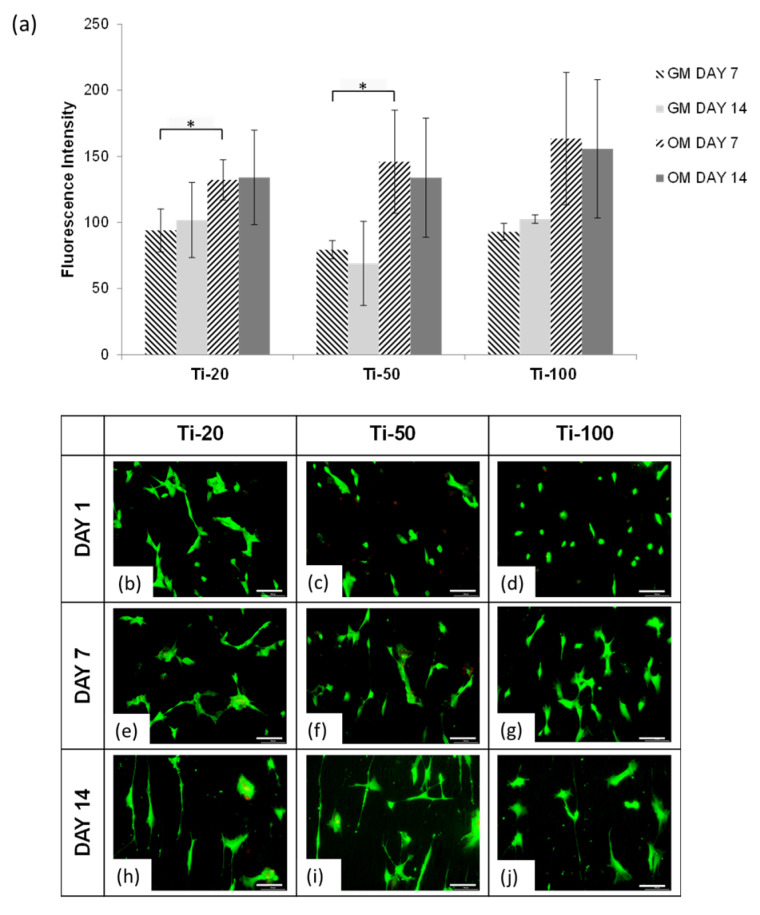
(**a**) AlamarBlue^TM^ metabolic activity of human mesenchymal stem cells (hMSCs) cultured on anodised titanium samples with respective pore diameter at Day 7 and 14 in growth medium (GM) and osteogenic medium (OM). Values are mean ± S.D., n = 4, * *p* < 0.05; (**b**–**j**) LIVE/DEAD cell viability microscopy of human mesenchymal stem cells (hMSCs) cultured on anodised titanium samples with respective pore diameter at Day 1, 7 and 14 in growth medium. Live cells were stained green and dead cells were stained red. Scale bar = 100 µm.

Figure 2a shows the AlamarBlue^TM^ metabolic activity of human mesenchymal stem cells (hMSCs) cultured on anodised titanium samples with respective pore diameter at Day 7 and 14 in growth medium (GM) and osteogenic medium (OM). The AlamarBlue^TM^ assay of hMSCs displayed an overall lower metabolic activity with GM on all the test samples. OM exhibited a higher proliferation rate, but not all significant when compared to GM at both collection days. In fact, the proliferation rate from Day 7 to Day 14 was retained or decreased in all samples.

Figure 2b–j shows LIVE/DEAD cell viability microscopy of human mesenchymal stem cells (hMSCs) cultured on anodised titanium samples with respect to pore diameter at Day 1, 7 and 14 in growth medium. The morphology of hMSCs cultured on test samples displayed a clear difference on Day 1, where Ti-20 samples showed the most extended cell adhesion (Figure 2b), followed by a partially extended cell body on Ti-50 samples (Figure 2c), and adhered cells without extension on Ti-100 samples (Figure 2d). Ti-20 samples presented the greatest cell density with overlapping structures and were polygonal shaped, while hMSCs on Ti-50 samples exhibited cell structures with wrapped edges, slight distortion and irregular cell shape. Ti-100 samples on Day 1 exhibited poor spreading of cells and rounded shape with weakly formed protein adsorption that facilitates cell focal adhesion, although the cell density was relatively high. Indications of cell spreading were presented on Day 7 on all the samples, with more sophisticated cell connecting structures on Ti-20 samples (Figure 2e). Ti-50 samples (Figure 2f) continued to reveal some dead cells similar to that of Day 1 with slight cell extension and overlapping structures, but with little intercellular communications. Filopodial extensions were displayed on Ti-100 samples on Day 7 (Figure 2g) following the cell network spreading and multiplication. On Day 14 in culture, all samples exhibited more elongated cells, especially on Ti-20 samples (Figure 2h), where more specialised cells can be detected. hMSCs on Ti-50 samples (Figure 2i) displayed overall elongated structures and connecting behaviours, but generally an inconsistent shape. The cells cultured on Ti-100 samples for 14 days revealed elongated growing structures with continual filopodial expansion around the cell structures. 

**Figure 3:**
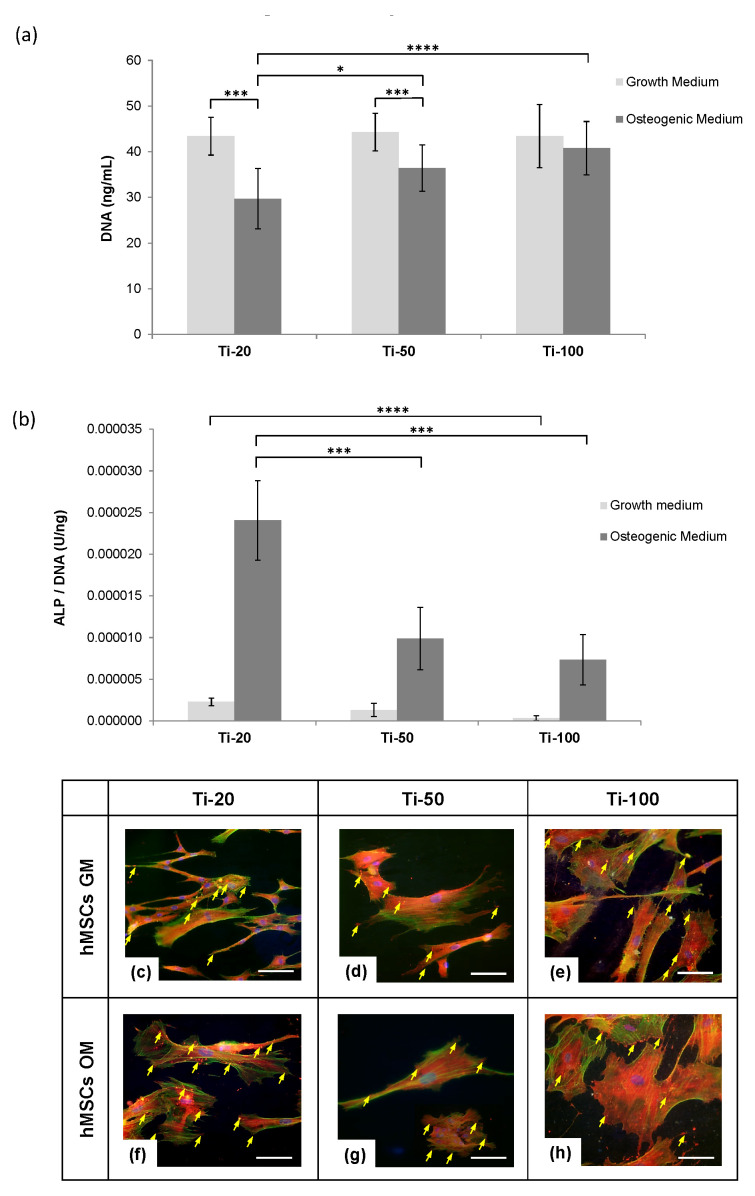
(**a**) DNA content of human mesenchymal stem cells (hMSCs) measured using PicoGreen assay, cultured on anodised titanium samples with respective pore diameter at Day 14 in growth medium (GM) and osteogenic medium (OM). Values are mean ± S.D., n = 4, * *p* < 0.05, *** *p* < 0.005, **** *p* < 0.001; (**b**) Normalised alkaline phosphatase (ALP) activity per DNA of human mesenchymal stem cells (hMSCs) cultured on anodised titanium samples with respective pore diameter at Day 14 in growth medium (GM) and osteogenic medium (OM). Values are mean ± S.D., n = 4, *** *p* < 0.005, **** *p* < 0.001; (**c**–**h**) Immunofluorescence microscopy with cell nuclei (blue), F-actin cytoskeleton (green) and vinculin (red) of human mesenchymal stem cells (hMSCs) cultured on anodised titanium samples with respective pore diameter at Day 14 in growth medium (GM) and osteogenic medium (OM). Yellow arrows indicate the focal adhesion sites. Scale bar = 100 µm.

The dsDNA quantification assay in Figure 3a displayed a consistent cell number on all the test samples over 14 days cultured with GM. An overall decreasing trend exhibited on cells cultured with OM when compared with GM in all test samples. Given the osteogenic growth factors in culture medium (OM), the cell numbers in Ti-20 samples were significantly lower than that of Ti-50 and Ti-100 samples. This phenomenon was commonly discovered in cultures that undergo a functional specialisation and significant differentiation. The cell number on Ti-100 samples was comparatively higher, but not significant to Ti-50 samples, signifying the continual growth of cells with these two sample types and the requirement for a further differentiation assay to quantify the differentiation rates among the test samples.

Figure 3b shows the normalised alkaline phosphatase (ALP) activity per DNA of human mesenchymal stem cells (hMSCs) cultured on anodised titanium samples for each pore diameter at Day 14 in growth medium (GM) and osteogenic medium (OM). An ALP assay corresponds to the marker for osteogenic differentiation. The normalised ALP activity showed a significant upregulation of ALP activity on Ti-20 samples in both GM and OM as compared with Ti-50 and Ti-100 samples. Most significantly, Ti-100 in GM condition exhibited the lowest osteogenic differentiation rate, with approximately 75% lesser than that of Ti-20 samples. It was also substantially significant that the hMSCs cultured in GM were approximately 10 times lower ALP activity in all test samples relative to their OM condition. Furthermore, Ti-50 and Ti-100 in OM condition exceeded more than 50% reduction in ALP levels as compared with Ti-20 samples.

Cytoskeleton could be seen to be well organised with defined actin stress fibres spread across the cytoplasm on Ti-20 samples (Figure 3c,f). In particular, for the OM condition in Figure 3f, the hMSCs displayed enhanced expression of thick, contractile stress fibres structures and notably more large focal adhesions at the cell membranes. However, both medium conditions of Ti-50 samples (Figure 3d,g) exhibited smaller focal adhesion plaques with less defined stress fibres compared to the other two sample types. It was also noted that the cells under OM conditions (Figure 3g) were single, with few focal contacts. The stress fibres assembly was extensive on Ti-100 samples in Figure 3e,h with well-spread structures but less organised focal adhesion sites. Actin fibres can be seen at the cell peripheries under OM condition (Figure 3h), but the cells were presented a stellate morphology with many uncoordinated focal adhesions.

### 3.3. Human Osteoblast Cells Responses

**Figure 4:**
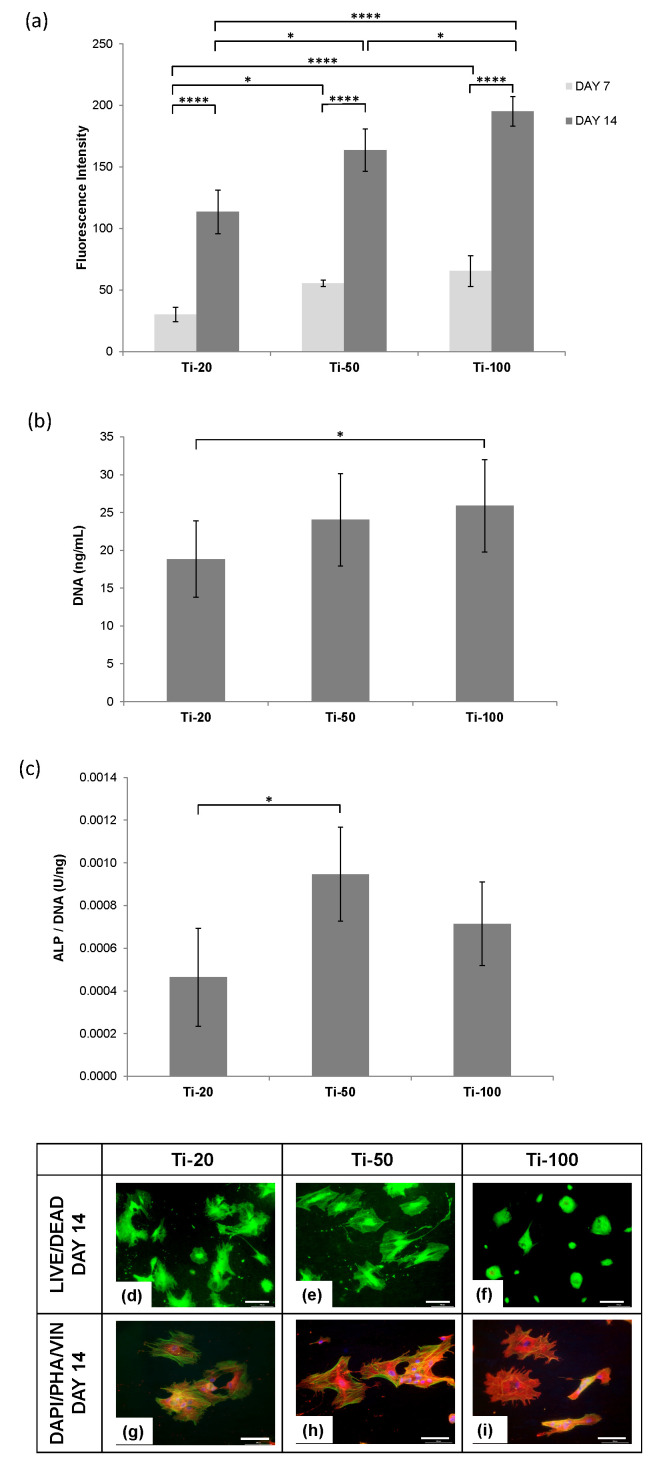
(**a**) AlamarBlue^TM^ metabolic activity of human osteoblasts (HOBs) cultured on anodised titanium samples with respective pore diameter at Day 7 and 14 in Osteoblast Growth Medium. Values are mean ± S.D., n = 4, * *p* < 0.05, **** *p* < 0.001; (**b**) DNA content of human osteoblasts (HOBs) measured using PicoGreen assay, cultured on anodised titanium samples with respective pore diameter at Day 14 in Osteoblast Growth Medium; (**c**) Normalised alkaline phosphatase (ALP) activity per DNA of human osteoblasts (HOBs) cultured on anodised titanium samples with respective pore diameter at Day 14 in Osteoblast Growth Medium. For (**b**,**c**) Values are mean ± S.D., n = 4, * *p* < 0.05; LIVE/DEAD cell viability microscopy (**d**–**f**) with green stained live cells and immunofluorescence microscopy (**g**,**h**) with cell nuclei (blue), F-actin cytoskeleton (green) and vinculin (red) of human osteoblasts (HOBs) cultured on anodised titanium samples with respective pore diameter at Day 14 in Osteoblast Growth Medium. Scale bar = 100 µm.

Figure 4a shows AlamarBlue^TM^ metabolic activity of human osteoblasts (HOBs) cultured on anodised titanium for the different pore diameters at Day 7 and 14 in Osteoblast Growth Medium. HOBs cultured in Osteoblast Growth Medium exhibited an approximately three-fold of metabolic activity from 7 to 14 days in culture, with Ti-20 samples significantly lower readings than that of other two test samples. Ti-100 exhibited the highest metabolic activity on both collection days, indicating that osteoblasts proliferated relatively better on Ti-100 samples, tightly followed by Ti-50 samples. Although on Day 14 there was a significant difference between Ti-50 and Ti-100 samples, Day 7 in culture was not significant between the two. The further functional assay should reflect the level of differentiation in correspondence with the lower proliferation rate of Ti-50 samples.

The dsDNA quantification assay in Figure 4b demonstrated that the HOBs cell number was the lowest on Ti-20 samples after two weeks in culture. Ti-50 and Ti-100 samples displayed similar cell number. Combining this finding with the lower proliferation rate of Ti-50 on Day 14, it was hypothesised that the HOBs underwent more differentiation activity from Day 7 to Day 14 as compared with Ti-100. An ALP activity assay was subsequently performed to test this hypothesis. 

The normalised ALP activity per DNA of HOBs in Figure 4c revealed that Ti-50 samples induced the most differentiation rate, closely followed by Ti-100 samples, and the lowest osteogenesis was discovered on Ti-20 samples. These findings are notably different to that of hMSCs culture, as shown in Figure 3b, indicating that stem cells behave differently from mature osteoblasts when cultured on the surface with different nanotube pore diameters. 

After two weeks in culture, the morphologies of HOBs were seen to be clearly different on all the test samples. As observed in Figure 4d, Ti-20 samples showed viable cells with distorted shapes and uncommon spreading morphology of typical osteoblasts. Ti-50 samples in Figure 4e, on the other hand, displayed a well spread and distinctive mature osteoblast shape with significant cell-cell and cell-material interactions. HOBs on Ti-100 samples exhibited wrapped cells with no sign of spreading and elongation, with small rounded and some big clumped morphologies, see Figure 4f. 

HOBs cultured on Ti-20 samples, shown in Figure 4g, presented well spread actin stress fibres but developed an irregular and migratory morphology with fewer and less well-defined focal contacts. The filopodia were observed on the cell to cell connection without significant projection onto the surface of the sample. Ti-50 samples (Figure 4h) supported HOBs with highly organised stress fibres with large, distinct focal adhesion sites. The cells expressed significant cellular connection and spreading morphology with a sophisticated cytoskeletal network. The Ti-100 samples in Figure 4i, in contrast, exhibited apoptotic morphology with a poorly defined cytoskeleton and minimal focal contacts.

## 4. Discussion

### 4.1. Human Mesenchymal Stem Cells

Nanotopographical features provided by the TiO_2_ nanotube mimic the hierarchical structure of natural bone extracellular matrix (ECM) more specifically, the protein fibres that made up the ECM, such as collagen fibrils and fibronectins [14,38,39]. These nanoscale features play a critical role in stem cell commitment and are involved in a complex pathway through the activation of transmembrane cell signalling proteins, more specifically, integrin receptors [40,41,42]. These integrin receptors act as primary receptors for ECM during the formation of focal adhesion complexes and could trigger intracellular signalling cascades into the cell nucleus via the contraction of actin stress fibres [16,43]. Hence, the integrin-mediated nanotopography interaction could dictate a series of major cellular activities, such as adhesion, proliferation, cytoskeletal reorganisation, motility, cell shape manipulation, differentiation, and apoptosis [15,22]. 

In the present study, it can be seen that hMSCs notably displayed a different morphology during the initial adhesion on the test samples. Ti-20 samples have provided a surface nanotopography that facilitates binding sites for focal contacts and protein adsorption. Compared with Ti-50 samples, Ti-100 evinced a rather significant difference in cell attachment morphologies, where the hMSCs were maintained in a rounded shape without stretching and spreading across the nanotube layer. These findings have confirmed the valid interdependency of nanoscale features and integrin clustering which dictates the cellular adhesion mechanism. Park et al. suggested that a lateral spacing of 15–30 nm has been found to be optimal for the activation of integrin gathering and focal contact formation, which further influence effective actin filament assembly, proliferation and mineralisation [18]; whereas nanotubes pore diameter over 70 nm inhibit the formation focal adhesions and impair the signal cascading pathways, which lead to cell quiescence or even programmed cell death [15,22,44]. Zhao et al. supported the argument by comparing 30 nm and 80 nm nanotube pore diameters on rat MSCs and concluded that the high-quality focal adhesion formation has resulted in considerable cell spreading, greater nodular ALP production and ECM mineralisation [19]. Bauer further elucidated that a significant reduction of stem cell activity was observed on nanotube diameters exceeding 50 nm [17]. 

On the other hand, contradicting results reported by Oh et al. and Zhao et al. indicated that 25–30 nm nanotube pore diameters had promoted considerable hMSCs adhesion, but a low differentiation rate, while a significant cell elongation possessed on 70–100 nm pore diameters have induced differentiation into osteoblast-like cells [14,24]. The findings in the present study supported the significant initial cell adhesion on smaller pore diameters and elongated cells on 100 nm nanotube diameters but are conflicting with the statement about the high differentiation rate as presented in the normalised ALP activity data; see Figure 3b. This discrepancy could be attributed to two critical factors, which are the normalisation assumptions and the additional treatment after anodisation. The differentiation expression levels were normalised to the positive control comprising hMSCs cultured on tissue culture plate with osteogenic medium and not the level of gene expression produced by each cell. It is worth noting that our findings suggest that Ti-20 samples have a significantly lower DNA number than Ti-50 and Ti-100 samples, but the former produced the highest level of ALP activity per DNA. The normalisation method employed by Oh et al. and Zhao et al. assumed the samples were had an equal amount of cells on each test sample, which can therefore lead to a potentially false impression. Moreover, the anodised samples were heat treated to transform the phase structure of the nanotube layer could also be a leading cause of this observation. The study brought a much complex mechanism into how cells react to nanotubular topography since it is well known that the phase difference could influence surface morphology and the degree of crystallinity could impact cellular behaviour and mineralisation ability in a synergistic manner [45]. In summary, while these studies discussed how a modified topography influenced osteogenic behaviour, they did not demonstrate the intracellular interactions towards the differences in surface nanotopography.

**Figure 5:**
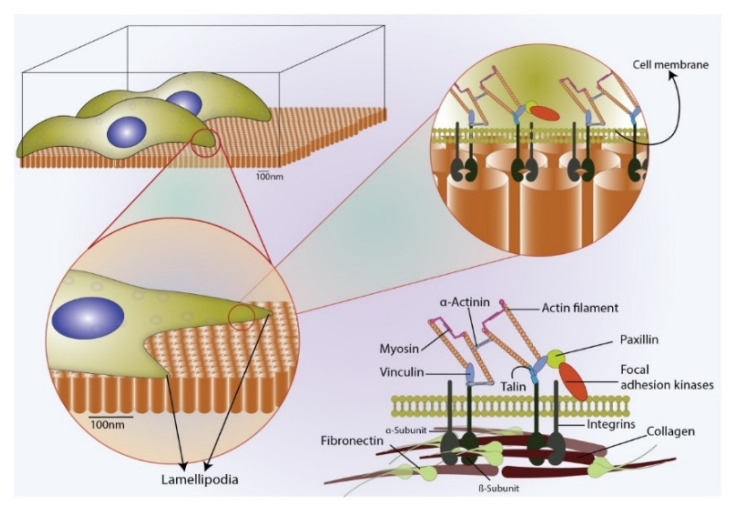
A schematic of cell-surface-induced mechanism, focal adhesion contact points for lamellipodia during cell adhesion on the nanotubular surface.

The bidirectional transmembrane communication of integrins is achieved through the heterodimeric protein receptors made up of α and β subunits, as illustrated in Figure 5. The extracellular domain then transmits the focal adhesion signals through the integrin signalling layer (consisted of focal adhesion kinase (FAK), paxillin and talin) to the force transduction layer (comprised of vinculin and zyxin), then further to actin regulatory layer that contains actin stress fibres [15]. There is an extensive amount of evidence suggesting that integrin-mediated adhesion signalling, and actin cytoskeleton contractility have a tight connection to mechanotransduction pathways [46,47,48,49]. Such a connection indirectly suggests that the size of focal adhesion assembly is a critical factor to osteo-specific differentiation than the number of adhesions [44,50]. The crosstalk between the extracellular receptor kinase (ERK) and tension-transmitted nucleoskeletal lamins regulates the cellular responses into a nanotopography-driven mechanosensitive signalling event [51,52]. The vinculin staining in the present study demonstrated that Ti-20 samples showed a significantly large anchorage plaque and the actin staining reflected strong cytoskeletal contractility in comparison to other two samples (Ti-50 and Ti-100), indicating the potential upregulation osteogenic phenotype through mechanosensitive events driven by activated focal adhesion assembly and reorganised actin stress fibres. The Ti-50 sample displayed a minimal amount of vinculin with poorly defined cytoskeletons, while Ti-100 samples exhibited a large amount of small vinculin and chaotically structured actin stress fibres which are thought to reduce the mechanotransductive signalling pathways and bone forming ability [44].

Park et al. suggested in their study that an approximately 20 nm spacing can foster closely packed integrins clustering that stimulates maximum integrin activation and promotes signal cascades to the nucleus for the upregulation of osteogenic pathways [17,22], whereas pore diameters above 30 nm would lead to unstable filopodia extensions and diameters of 100 nm could substantially impede adhesion, mobility and differentiation [18,19]. The findings of the current study are consistent with those reported in earlier studies, wherein the highest ALP production was revealed in Ti-20 samples, and the lowest was found in Ti-100 samples using both GM and OM conditions. The immunostaining microscopy also substantially exhibited the bone-like remodelled structures on Ti-20 samples as compared with the other two test samples. Taken together the evidence from other earlier studies [17,19,22], it is concluded that TiO_2_ nanotube with pore diameters of approximately 20 nm could synergically induce hMSCs osteogenesis and osteoprogenitor remodelling. 

### 4.2. Human Osteoblasts

Osteoblasts responses were rather conflicting in the literature, with some agreed on better cell viability found on nanotube less than 30 nm pore diameter (Group 1) [31,32] and some suggested greater osteoblastic functions found on pore diameter between 30 nm and 50 nm (Group 2) [13,26,27,28,29,30], but only a few have proposed high osteogenic responses on the nanotube layer with pore diameter over 70 nm (Group 3) [33,35]. It is worth noting that the reports published in Group 1 and Group 3 were limited to only cell adhesion percentage [31], cell viability and proliferation rate [32], with a limited comparison between nanotube ranges [33] and generalised assumptions on osteoblastic responses [35], these studies were lacking a clear reasoning on osteogenic differentiation in general. On the other hand, studies reported in Group 2 were more convincing due to the involvement of a wider nanotube diameter range and empirical data to elucidate the osteogenic behaviours of osteoblastic cells cultured on the nanotubular layer. Although much effort has made in investigating these events, there was no universal agreement established regarding osteoblast responses on suitable nanotube diameter range, and the clear discrepancy with findings observed in the mesenchymal stem cells was never identified and discussed. 

It was commonly recognised that nanotube pore diameter greater than 70 nm would reduce cellular activity and trigger cell apoptosis. In this study, a clear illustration of programmed death was visualised in Ti-100 samples (Figure 4i). Although the improved hydrophilic character of Ti-100 samples was able to retain more HOBs onset initial seeding [26] but the dominating topography did not allow the formation of quality focal contacts that are responsible for inducing the assembly of actin filaments and signal cascading to the cell nucleus [22]. The impairment of intracellular signalling mechanism following the failure of integrin anchoring have proven to be the leading factor for cell quiescence or more severely, programmed cell death via apoptosis [15,16]. 

A controversy, however, arises between the nanotube pore diameter preferred by hMSCs and HOBs. The findings in this study may give a clearer answer to this discrepancy. The hMSCs significantly showed a greater differentiation rate coupled with a notably distinctive morphology and focal contacts that induce higher osteogenesis on Ti-20 samples, while HOBs have displayed strong differentiation expressions and bone cell maturation towards Ti-50 samples. To explain these events, the intracellular and extracellular mechanisms correlating the bidirectional signalling integrins have to be understood. Dalby et al. described the role of the cell as a part of integrin-matrix interaction in a close analogy to a tent: “the larger the tent (cell), the larger the pegs (integrin clusters) would need to be to cope with the tension applied to the guy ropes to provide adequate integrity” [15]. As hMSCs are 10–20 µm in diameter [53] and HOBs are 20–30 µm in diameter [54], it is hypothesised that HOBs required larger adhesions in comparison with hMSCs [55,56]. 

In our investigations, we found that the interwall dimension between the inner pore and the inner pore of the closest pore, derived from interpore distance subtract pore diameter of Ti-20 samples, were approximately 2.7 nm, Ti-50 nm samples were approximately 15.5 nm, and Ti-100 samples were around 34.0 nm. Due to the larger cell size of HOBs, it is deduced that the greater adhesions and the activation of osteogenic differentiation through intracellular cascading were induced by the interwall dimension of Ti-50 samples, which correlates well with the earlier studies suggested that 15–30 nm spacing could result in compact clustering of integrin receptor molecules [17,18,22]. Furthermore, another seminal paper on nanotopography-driven differentiation also suggested that disordered nanoscale pits randomly displaced by up to 50 nm could trigger osteogenic differentiation as compared to aligned patterns, even without the presence of biochemical osteoinductive factor [57]. This finding corroborates with the present results, which involved HOBs cultured in Osteogenic Growth Medium without the addition of soluble osteogenic factors, have supported the upregulation of ALP production on Ti-50 samples as compared with Ti-20 samples. The immunostaining microscopy further displayed the osteoblastic maturation morphology on Ti-50 samples than the other two samples. Concluding the above observations, it is substantiated that the complex interplay between the interconnected mechanotransductive effects of nanotopography-mediated integrins clustering and cytoskeletal tension could impact osteoblastic differentiation in a significant manner [16]. 

## 5. Conclusions

While existing research has been carried out in the literature, there has been no single systemic study to date which succinctly compares the response of human stem cells and osteoblasts side by side with a range of TiO_2_ nanotube pore diameters using controlled experiments in a single laboratory. This study showed the interrelationship between cellular behaviours and nanotopography, and has confirmed the hypotheses made previously: (i) a 20 nm nanotube pore diameter is preferred by human mesenchymal stem cells (hMSCs) for the induction of osteogenic differentiation, (ii) 50 nm nanotubular structures are favourable by human osteoblasts (HOBs) for osteoblastic maturation; and (iii) a nanotube pore diameter of 100 nm acts to impede osteogenesis and impair intracellular signalling which potentially leads to programmed cell death. These new findings have clarified the underlying mechanisms of the transmembrane bidirectional pathways and could offer new insights into discrete nanotopographical implant surface tailoring for targeted cells and tissues specialisation. Although there is much more work to be done to verify the efficacy of titania nanotube to be used in the clinical and surgical arena, this research creates a paradigm for future studies on the evolution of orthopaedics in the area of targeted osseointegration through tailored innovative nanotopography approach. 

Though ALP activity is adequate to assess the osteogenic markers of the cells, it is recommended to include more functional assays in future studies to provide a better overview of the cell-nanotopography interactions. These complementary assays should include the investigation of the extracellular matrix (ECM) proteins released by the cells by enzyme-linked immunosorbent assay (ELISA), or through polymerase chain reaction (PCR) test, for evaluating the expression of protein markers involved in osteogenesis, such as COL-1, osteopontin and osteocalcin. Addition research shall also include the concentration of collagen and fibrinogen to conclude the relationship between the distance between nanostructure and cell behaviours. As human fetal osteoblasts typically proliferate at 34 °C and differentiate at 39 °C [58,59]. The future study shall also consider the transfection ability of the temperature-sensitive gene in the cells corresponding to the nanotube diameters. Furthermore, high magnification visualisation of filopodial growth into the nanotubes could also be an interesting exploration area to understand how cells adapt and integrate with the nanoscale architectures to create an interlocking cell structure. Together with these investigations, a better comprehension of the cell-nanotopography interactions and mechanisms could be achieved to offer new insights into tailoring cellular osteogenic behaviours and bone tissues specialisation through controlled surface topography.

Although the present study has successfully demonstrated the integrin-nanotopography and intracellular interactions on osteogenic induction, it has certain limitations concerning the clinical application of the research especially when the surface of titania nanotube to be used in a real complex and dynamic environment. While it is understood that different cell lineages could respond differently on the surface with nanoscale features, further study could include multi-levelled nanotube created by two-step anodisation [32] and surface with disordered nanotopography comprising multi-nanoscale ranges [57], to facilitate favourable responses from different cell types using these multiscale biofunctionalised substrates. Meanwhile, titanium alloys are getting more attention due to their excellent strength-to-weight ratio and lower elastic modulus than pure titanium, making them even more suitable for use as implant materials [60,61]. Thus, further work should focus on anodising nanotubes on a variety of alloyed titanium for biological applications. When implants come into contact with blood during insertion and implant placement, the activation of platelet adhesion may cause thrombosis and implant failure [62,63]. Hence, implant haemocompatibility is of importance to prevent platelet aggregation. Many studies in recent years focus on implant surface modification to improve haemocompatibility [64,65,66]. Among them, Movafaghi et al. reported superhemophobic titania nanotubes morphologies via electrochemical anodisation could provide considerable haemocompatibility characters [65]. Hence, further study in nanotubular fabrication could emphasise not just on osseointegration, but also the haemocompatibility of the modified surface.
